# Medical Progress

**Published:** 1855-10

**Authors:** 


### MEDICAL PROGRESS.

*Reese's Medical Gazette* for September has a new feature in medical journalism, "*Selections from favorite prescriptions of living American practitioners.*" We hope this will be continued. Medical science has been greatly retarded by its awe of the old and its fear and contempt of the new. It is a Herculean labor to get a physician to try a new remedy, unless "*authority*" sanctifies it. Thus it is, that the so-called *New Schools*, *Reformed Schools*, have gained large advantages over scientific medicine. They are willing to try anything, and to employ in their practice whatever remedy seems, from actual experience, to be worthy of confidence. It is the only rational practice of medicine, and for the following reasons.

1. Climate is constantly changing.
2. The constitutions of men are constantly changing.
3. The habits of society are constantly changing.
4. The circumstances and conditions of domestic life are constantly changing.

Such being the case, that practitioner cannot command success, who administers to-day, the same remedy for the same symptom, which he did twenty years ago. Every observant physician knows that the types of disease vary from year to year, and he is the most successful man who earliest notices that change, and judiciously adapts his remedies to it. This is the key to successful practice everywhere. This gives "*eminence*" to men of the time, and we want their experience and "*prescriptions.*"

